# Profile of Innate Immunity in Gilthead Seabream Larvae Reflects Mortality upon Betanodavirus Reassortant Infection and Replication

**DOI:** 10.3390/ijms23095092

**Published:** 2022-05-03

**Authors:** Miguel Ángel García-Álvarez, Marta Arizcun, Elena Chaves-Pozo, Alberto Cuesta

**Affiliations:** 1 Immunobiology for Aquaculture Group, Department of Cell Biology and Histology, Faculty of Biology, Regional Campus of International Excellence “Campus Mare Nostrum”, University of Murcia, 30100 Murcia, Spain; miguelangel.garciaa1@um.es; 2 Oceanographic Center of Murcia, Spanish Institute of Oceanography, Spanish National Research Council (IEO-CSIC), Carretera de la Azohía s/n. Puerto de Mazarrón, 30860 Murcia, Spain; marta.arizcun@ieo.es (M.A.); elena.chaves@ieo.es (E.C.-P.)

**Keywords:** nodavirus, reassortants, virus, gilthead seabream, larvae, immunity

## Abstract

Historically, gilthead seabream (*Sparus aurata*) has been considered a fish species resistant to nervous necrosis virus (NNV) disease. Nevertheless, mortality in seabream hatcheries, associated with typical clinical signs of the viral encephalopathy and retinopathy (VER) disease has been confirmed to be caused by RGNNV/SJNNV reassortants. Because of this, seabream larvae at 37 and 86 days post-hatching (dph) were infected by immersion with RGNNV/SJNNV and SJNNV/RGNNV reassortants under laboratory conditions, and mortality, viral replication and immunity were evaluated. Our results show that gilthead seabream larvae, mainly those at 37 dph, are susceptible to infection with both NNV reassortant genotypes, with the highest impact from the RGNNV/SJNNV reassortant. In addition, viral replication occurs at both ages (37 and 86 dph) but the recovery of infective particles was only confirmed in 37 dph larvae,; this value was also highest with the RGNNV/SJNNV reassortant. Larvae immunity, including the expression of antiviral, inflammatory and cell-mediated cytotoxicity genes, was affected by NNV infection. Levels of the natural killer lysin (Nkl) peptide were increased in SJNNV/RGNNV-infected larvae of 37 dph, though hepcidin was not. Our results demonstrate that the seabream larvae are susceptible to both NNV reassortants, though mainly to RGNNV/SJNNV, in an age-dependent manner.

## 1. Introduction

Marine aquaculture is a continuously expanding sector, which must deal with numerous pathogens that cause economic losses. Among these pathogens, nervous necrosis virus (NNV) is one of the most threatening viruses, affecting to more than 170 marine teleost fish species, and being the causative agent of the viral encephalopathy and retinopathy (VER) disease. NNV is a 25–30 nm icosahedral virus, non-enveloped, whose genome consists of two molecules of singled-stranded positive-sense RNA, called RNA1 and RNA2 [1]. The RNA1 segment codifies for the RNA-dependent RNA polymerase (RdRP) whereas the RNA2 segment encodes the capsid protein (CP). Additionally, a third RNA segment, RNA3, is synthesized during viral replication due to the cleavage of RNA1, producing B1 and B2 proteins, the latter being needed for the repression of cellular RNA interference in infected cells [2,3]. Traditionally, NNV has been classified into four genotypes, based on a variable region of the RNA2 segment named T4: striped jack nervous necrosis virus (SJNNV), red-spotted grouper nervous necrosis virus (RGNNV), barfin flounder nervous necrosis virus (BFNNV) and tiger puffer nervous necrosis virus (TPNNV). Nonetheless, an additional genotype has been accepted, named as turbot nodavirus (TNV) [4]. However, recent studies have indicated the existence of two natural genotype reassortants: the first one, isolated from European sea bass (*Dicentrarchus labrax*) and known as SJNNV/RGNNV [5], and the second one, isolated from gilthead seabream (*Sparus aurata*) and Senegalese sole (*Solea senegalesis*), known as RGNNV/SJNNV [6]. These are named for the presence of the paternal donor RNA1 and RNA2 segment, accordingly. Analysis of the RNA fragments of the reassortants has indicated that they show differences from the parental fragments, those differences being more pronounced in RdRP than in CP, which is surprising because structural proteins have a higher rate of variability than non-structural proteins [6]. However, the ability of fish nodavirus to infect different species is controlled by CP [7]. Additionally, RNA1 play a key role in the regulation of viral replication at different temperatures [8]. Therefore, further knowledge of these reassortants at the genetic and molecular levels is mandatory.

Gilthead seabream and European sea bass are the most farmed fish species in the Mediterranean area. Traditionally, European sea bass has been considered a very susceptible species to RGNNV, while gilthead seabream acts as a resistant species [9,10,11]. Surprisingly, in recent years, natural outbreaks have been described in hatcheries with seabream larvae showing typical symptoms of VER disease, including discoloration of the skin, abnormal swimming behavior and cytoplasmic vacuolation of nervous tissues, the brain and retina. The causes of these outbreaks were genetically identified as RGNNV/SJNNV reassortants [12,13,14]. Moreover, the RGNNV/SJNNV reassortant genotype seems to exhibit a higher tropism to very early larvae stages of seabream, with higher virulence and mortality rates than in later stages of development [15]. On the contrary, European sea bass have not shown evident clinical signs upon RGNNV/SJNNV infection [4]. Nevertheless, both fish species can present persistent infection and may act as asymptomatic carriers of the NNV reassortants [14]. As a matter of fact, in vitro studies carried out with RGNNV/SJNNV strains point out that its optimal temperature is similar to that of RGNNV, around 25 °C [8]. A transcriptomic analysis of gilthead seabream larvae infected with RGNNV/SJNNV revealed an up-regulation of genes related to heat-shock proteins, while those genes related to immunity, including interferon (IFN) and other antiviral pathways, among others, were down-regulated [16]. In contrast, no information is available about the infective capacity of SJNNV/RGNNV genotypes. Differences in infectivity, disease outcomes or host–viral interactions between NNV reassortants and the new susceptible hosts are needed to unravel the potential future problems in the sector and how to solve them.

Thus, the main objective of this study was to provide evidence for the infectivity of both RGNNV/SJNNV and SJNNV/RGNNV reassortant genotypes in gilthead seabream larvae at two different ages, as well as the regulation of gene expression, in order to better understand the host–NNV interactions and their potential negative effects on fish farms in the Mediterranean Sea.

## 2. Results

### 2.1. Both NNV Reassortants Produce Mortality in Seabream Larvae

**Figure 1:**
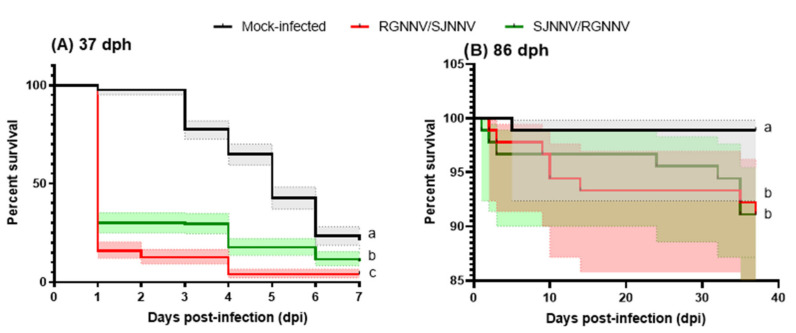
Nodavirus (NNV) reassortants produce mortality in gilthead seabream larvae. Seabream larvae at 37 (**A**) or 86 days (**B**) post-hatching (dph) were infected by immersion with 10^4^ TCDI_50_/_mL_ of RGNNV/SJNNV or SJNNV/RGNNV reassortants and mortality recorded daily. The control was a mock-infected group. Kaplan–Meier survival curves showing the mean percentage of seabream larvae survival (solid line; *n* = 3) and the 95% confidence intervals (in shadows). Different letters denote statistical differences according to the Log-rank (Mantel–Cox) test (*p* < 0.05).

The transfer of seabream larvae from an optimal hatching environment to the infection conditions, including the immersion infection process and the stabling in the infection tanks at high densities, lead to a great mortality in 37 days post-hatching (dph) larvae from 2 days post-infection (dpi) onwards, as seen in the mock-infected controls (Figure 1A), probably due to suboptimal and stress conditions. However, infection of 37 dph larvae with the NNV reassortants resulted in a marked and drastic drop in fish survival during the first 24 h post-infection (Figure 1A). At the end of the trial, the larvae survival rate was 21% in the mock-infected group while this was reduced to 4 and 10% in the RGNNV/SJNNV (*p* < 0.0001) and SJNNV/RGNNV (*p* < 0.0001) reassortant infection groups, respectively, both showing significant differences with respect to the mock-infected group, and between them (*p* < 0.0001). Regarding the 86 dph larvae, the infection process was not stressful for the larvae and few mortalities were observed in the mock-infected group (Figure 1B). Larvae infected with RGNNV/SJNNV or SJNNV/RGNNV reassortants showed a significant reduction in survival with respect to the control group (98.8%) reaching 92% (*p* = 0.0173) and 91% (*p* = 0.0172), respectively (Figure 1B) though no statistical differences between them (*p* = 0.9939) were observed.

### 2.2. NNV Reassortants Replicate and Produce Infective Particles in Seabream Larvae

**Figure 2:**
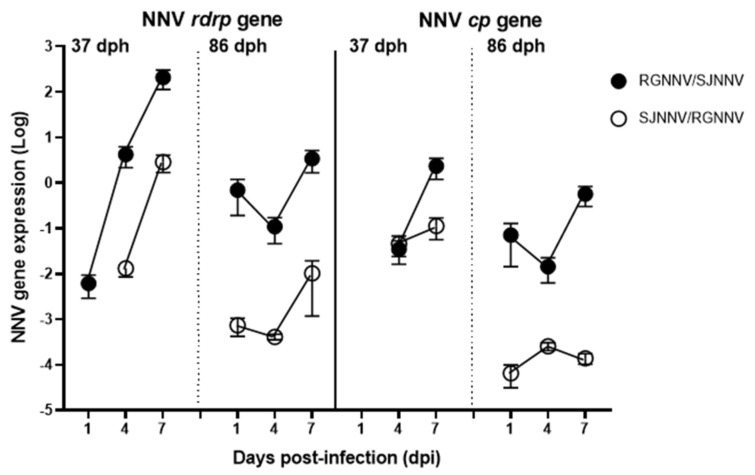
Nodavirus (NNV) reassortants replicate in gilthead seabream larvae. Seabream larvae at 37 or 86 days post-hatching (dph) were infected by immersion with 10^4^ TCDI_50_/_mL_ of RGNNV/SJNNV or SJNNV/RGNNV reassortants. The control was a mock-infected group. At 1, 4 or 7 days post-infection (dpi) individual larvae (*n* = 5) were sampled and the transcription of the viral RNA-dependent RNA polymerase (*rdrp*) and coat protein (*cp*) genes analysed by real-time PCR. Data represent the mean relative gene expression ± SEM.

The mRNA levels of the two viral genes, *rdrp* and *cp*, were evaluated to confirm viral infection and potential replication. First, we compared qPCR efficiency for the detection of each gene in the two reassortants in order to make comparisons. Thus, efficiencies of *rdrp* primers for RGNNV/SJNNV were 109%, and 106% for SJNNV/RGNNV, while the efficiency of *cp* primers was 102% for both NNV reassortants, allowing proper detection and comparisons among them. NNV genes were never detected in mock-infected larvae. Comparing NNV gene transcription, we observed that: (i) NNV detection is higher in 37 dph than in 86 dph larvae, except the *cp* gene at 1 dpi; (ii) NNV genes increase with the infection time; (iii) NNV *rdrp* levels were mostly higher than NNV *cp* ones; and (iv) NNV detection was always higher in seabream larvae infected with RGNNV/SJNNV than with SJNNV/RGNNV (Figure 2). Focusing on 37 dph whole larvae, the presence of *rdrp* and *cp* transcripts was firstly detected at 1 and 4 dpi with RGNNV/SJNNV and SJNNV/RGNNV genotypes, respectively. The NNV *rdrp* expression in the RGNNV/SJNNV-infected larvae increased by more than 4 logs, reaching the maximum levels at 7 dpi. In the anterior third of the 86 dph larvae, the presence of both genes was detected at 1 dpi for the two reassortants, reaching maximum expression at 7 dpi, though increment over time was limited to around 1 log.

**Figure 3:**
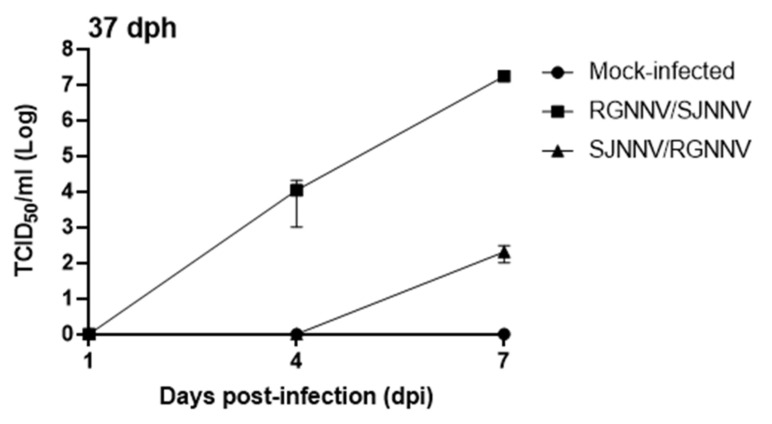
Infective nodavirus (NNV) particles are isolated from seabream larvae. Seabream larvae at 37 days post-hatching (dph) were infected by immersion with 10^4^ TCDI_50_/_mL_ of RGNNV/SJNNV or SJNNV/RGNNV reassortants. The control was a mock-infected group. At 1, 4 or 7 days post-infection (dpi) pooled whole larvae (*n* = 3) were homogenized and the viral titer determined in E-11 cell cultures. Data are shown as mean titer ± SEM.

To further confirm this, we evaluated the viral load by the isolation of infective particles using E-11 cell cultures. Thus, in 37 dph seabream larvae we were able to isolate and titrate NNV in pooled specimens infected with RGNNV/SJNNV after 4 and 7 days or with SJNNV/RGNNV after 7 days (Figure 3). In the case of RGNNV/SJNNV-infected larvae, the increase in the viral load from 4 to 7 dpi was higher than 3 logs. Parallel to the gene expression, viral load increased with the infection time, and was higher in RGNNV/SJNNV- than in SJNNV/RGNNV-infected larvae. However, in 86 dph larvae, though we could not test this at 1–7 dpi due to the lack of samples, no virus was isolated for both reassortant genotypes at 15 dpi (data not shown).

### 2.3. Nkl Protein Is Increased in 37 dph Larvae Infected by SJNNV/RGNNV

**Figure 4:**
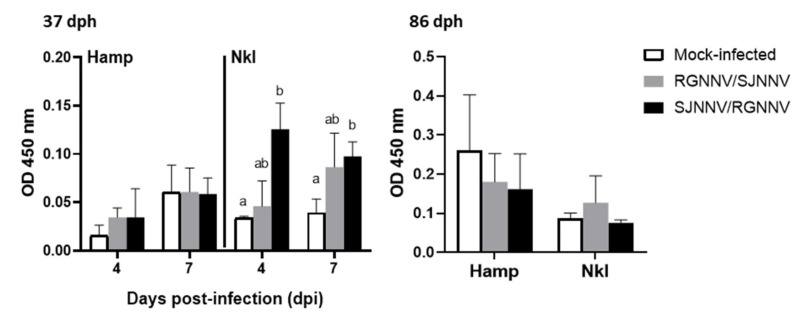
Early seabream larvae show increased levels of NK-lysin (Nkl). Protein levels of Nkl and hepcidin antimicrobial peptide (Hamp) in 37 and 86 days post-hatching (dph) seabream larvae infected with 10^4^ TCDI_50_/_mL_ of RGNNV/SJNNV or SJNNV/RGNNV reassortants at 1, 4 and 7 days post-infection (dpi). The control was a mock-infected group. Data represent the mean ± SEM (*n* = 3). Letters indicate statistical differences between treatments by one-way ANOVA (*p* < 0.05) followed by Tukey’s post-hoc analysis.

To investigate the involvement of the innate immune system during the infection with the NNV reassortants, the protein levels of two AMPs, Nkl and Hamp, were studied by an indirect ELISA. Our results show different levels of both proteins in 37 and 86 dph larvae, being higher in the oldest larvae (Figure 4). Furthermore, Hamp levels were not altered by infection with NNV reassortants while Nkl protein levels presented a significant increase in 37 dph larvae infected with SJNNV/RGNNV at 4 and 7 dpi (Figure 4). In 86 dph larvae, Hamp and Nkl levels were not significantly altered by NNV infection at 15 dpi.

### 2.4. Larval Age Is More Important than NNV Reassortant or Infection Time Factors

Once we had corroborated the fact that both viral genotypes are able to infect and cause mortality in larvae at different development stages, we wanted to study the relationship between all the parameters determined (survival, viral replication and load as well as protein and mRNA levels) at 1, 4 and 7 dpi. We first evaluated all the parameters by ANOVA (analysis of the variance) and PCA (principal components analysis) to identify the most important factor (age, infection time and NNV genotype). Thus, we found that the age factor has the strongest impact, with a significant effect in 11 out of the 13 genes evaluated and in the larval survival, followed by the infection time, and finally the NNV reassortant (Table 1). Viral load, *rdrp* transcription and survival show significance for all the factors alone or their combinations. Interactions between the two or the three factors were also observed in some immune genes (Table 1).

**Figure 5:**
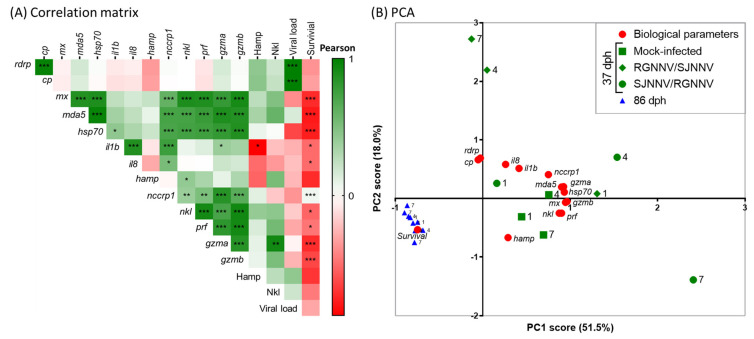
Different gene correlations and age clusters are observed in seabream larvae upon reassortant infection. Correlation (**A**) and principal components analysis (PCA) (**B**) of the mean gene transcription, peptide levels, viral load and survival in 37 and 86 days post-hatching (dph) seabream larvae infected by immersion with 10^4^ TCDI_50_/_mL_ of RGNNV/SJNNV or SJNNV/RGNNV reassortants at 1, 4 and 7 days post-infection (dpi). (**A**). Mean values of the parameters were correlated and the Pearson index presented in a heatmap (*, *p* < 0.05; **, *p* < 0.01; ***, *p* < 0.001). (**B**). PCA score plot, showing two components explaining 71.5% of the variance, based on the mean values of biological parameters (gene expression and survival). Colors and shapes denote the infection group and numbers the infection time. *rdrp*, NNV RNA-dependent RNA polymerase; *cp*, NNV coat protein; *mx*, interferon-induced GTP-binding protein Mx; *mda5*, melanoma differentiation-associated protein 5; *hsp70*, heat-shock protein 70; *il1b*, interleukin 1 beta; *il8*, interleukin 8; *hamp*, hepcidin antimicrobial peptide; *nccrp1*, non-specific cytotoxic cell receptor protein 1; *nkl*, NK-lysin; *prf*, perforin; *gzma*, granzyme A; *gzmb*, granzyme B.

We then evaluated their correlations. Both viral genes show strong cross-correlation between them and with the viral load (Figure 5A) (Pearson index > 0.99, *p* < 0.000) but not with the immune genes. Among the immune-related genes, antiviral (*mx* and *mda5*), stress (*hsp70*) and CMC response (*nccrp1*, *prf*, *nkl*, *gzma* and *gzmb*) markers show a strong correlation each other (Figure 5A). Inflammatory genes (*il1b* and *il8*) show good cross-correlation between them and with the *nccrp1*, while the antimicrobial peptide *hamp* only correlates with the other AMP, *nkl*. Hamp levels were negatively correlated with the transcription of the inflammatory *il1b* gene whilst Nkl levels positively correlated with the *gzma* gene expression (Figure 5A). Interestingly, viral load and survival show negative correlation with most of the immune genes analysed.

PCA analysis reinforced the above observations and identified two components explaining 71.5% of the variance (Figure 5B). The first component of the analysis (PC1 = 53.5%) increases with increasing *mx*, *mda5*, *hsp70*, *prf*, *gzma*, *gzmb*, *nkl* and *nccrp1* gene expression (Figure 5B, Supplementary Table S1), which agrees with the great and positive correlations observed among them (Figure 5A). The second component of the analysis (PC2 = 18%) increases with increasing viral and inflammatory genes, though decreasing *hamp* gene expression (Figure 5B, Supplementary Table S1). Moreover, the two components decrease with decreasing survival. In addition, when data were clustered by the main factor, larval age, we detected that all the genes from 86 dph larvae clustered very tightly and apart from those of 37 dph, which showed great variability among reassortant virus and infection times.

### 2.5. Immune Genes Are Differently Modulated by NNV Reassortants

**Figure 6:**
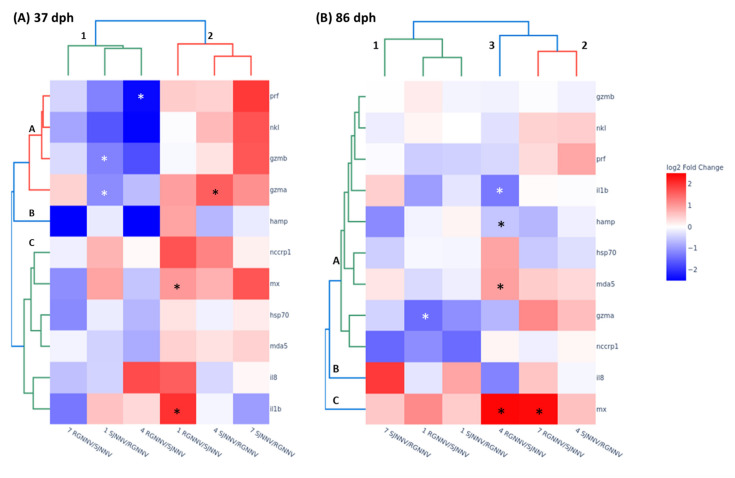
Seabream larvae of different ages show great variation in immune-related gene expression upon reassortant nodavirus (NNV) infection. Heatmap and two-way hierarchical clustering analysis (Euclidean method) of differentially expressed genes in 37 (**A**) and 86 (**B**) days post-hatching (dph) seabream larvae infected by immersion with 10^4^ TCDI_50_/_mL_ of RGNNV/SJNNV or SJNNV/RGNNV reassortants at 1, 4 and 7 days post-infection (dpi). Data are shown as mean of log2 fold change (*n* = 5) with respect to the mock-infected group. Asterisks (*) represent statistical differences between control and NNV-infected groups according to a *t*-Student test (*p* < 0.05). Letters inside figures (A, B, C) denote clusters for genes and numbers inside figures (1, 2, 3) denote clusters for samples. *mx*, interferon-induced GTP-binding protein Mx; *mda5*, melanoma differentiation-associated protein 5; *hsp70*, heat-shock protein 70; *il1b*, interleukin 1 beta; *il8*, interleukin 8; *hamp*, hepcidin antimicrobial peptide; *nccrp1*, non-specific cytotoxic cell receptor protein 1; *nkl*, NK-lysin; *prf*, perforin; *gzma*, granzyme A; *gzmb*, granzyme B.

Having evidenced the great differences between larval ages, we present the transcription fold change in two age-separated heatmaps with hierarchical clustering (Figure 6). Concerning the results in 37 dph larvae, clustering analysis yields two groups of samples with similar expression profiles: cluster 1, comprising seabream larvae infected with RGNNV/SJNNV at 4 and 7 dpi and with SJNNV/RGNNV at 1 dpi, and cluster 2 with those infected with SJNNV/RGNNV at 4 and 7 dpi and with RGNNV/SJNNV at 1 dpi (Figure 6A). Interestingly, most genes of cluster 1 were down-regulated whilst in cluster 2 were up-regulated. This indicates a relationship between the onset of the infection with RGNNV/SJNNV genotype and the mid/late stages for SJNNV/RGNNV, and *vice-versa.* Genes are also grouped into two clusters: A and C, with *hamp* gene as an outlier (B). Cluster A contains genes related to the cell-mediated cytotoxicity while cluster C is represented by genes related to antiviral, proinflammatory and stress responses, which is also observed in the correlation matrix (Figure 5A).

Hierarchical clustering analysis of 86 dph larvae (Figure 6B) reveals two clusters and one outlier due to its gene expression profile. Thus, cluster 1 includes seabream infected with RGNNV/SJNNV at 1 dpi and with SJNNV/RGNNV at 1 and 7 dpi, and cluster 2 comprises those infected with SJNNV/RGNNV at 4 dpi and with RGNNV/SJNNV at 7 dpi, with RGNNV/SJNNV at 4 dpi being the outlier (3). Regarding genes, they were divided into three clusters: A, comprising most of the genes, and two outliers, B (*il8*) and C (*mx*). Clusters 1 and 2 present similar expression for most of the seabream genes except a down-regulation of *gzma* by RGNNV/SJNNV at 1 dpi and an up-regulation of the *mx* gene by RGNNV/SJNNV at 7 dpi. However, the outlier cluster 3 includes more regulated genes than any other, with a significant decrease of *il1b* and *hamp* genes and an increase of *mda5* and *mx* genes (Figure 6B). Therefore, the infection with both reassortants was similar at the beginning of the infection, but once it had reached the fourth day, there was no relation between both genotypes, presenting a greater transcriptomic response to the RGNNV/SJNNV genotype, higher at 4 than at 7 dpi.

## 3. Discussion

The emergence of nodavirus reassortant genotypes has provided a new focus of attention to prevent and combat disease transmission in fish farms, because they present different patterns of pathogenicity and exhibit tropism towards new species. The most prominent is the case of gilthead seabream, which allows the replication of RGNNV and SJNNV genotypes but is refractory to developing the VER disease and mortalities [10,17]. However, recently, different natural outbreaks and laboratory trials have well documented the infective capacity of RGNNV/SJNNV reassortants in gilthead seabream during early development [13,14,15]. A clear relationship between the age of the individuals and the mortality rates has been established. Thus, nearly 100% mortality is observed in early larval stages (17–35 dph) and almost null in older stages [13,14,15]. Strikingly, these reassortants produce low mortality rates in European sea bass (*Dicentrarchus labrax*) [14], a well-known and very susceptible species to canonical RGNNV strains. In this study, we determined the infection capacity under laboratory conditions of the RGNNV/SJNNV genotype in 37 and 86 dph seabream larvae, supporting, but also expanding on, previous findings. Therefore, our study also confirmed, for the first time, the infection capability of the SJNNV/RGNNV genotype against gilthead seabream. It is known that larvae stages are very sensitive to environmentally-induced stress, which induces a passive behavior that prevents the efficient hunting of live prey [18]. In our experiments, and contrary to previous studies [15], we transferred the larvae to an infection unit with constraint measures, to avoid viral escape to the environment, and then bath exposed them to NNV at high densities using standard protocols. Therefore, it is likely that this stress resulted in starvation of the larvae due to impaired depredatory behavior and a subsequent mortality at middle/long term [18], as observed in mock-infected fish. Though this could be seen as an experimental limitation in the laboratory, it might represent more realistic situations in the hatcheries due to punctual technical/environmental/managing perturbations. However, viral infection of the control group was discarded since viral RNA, infective particles or alteration of the antiviral gene markers were never observed in mock-infected fish. Therefore, the differences in mortality between controls and infected groups were due to NNV infection even when suboptimal culture conditions during the infection trial altered the survival of the mock-infected fish.

After taking the experimental limitations into account, our results confirmed that both RGNNV/SJNNV and SJNNV/RGNNV reassortants induced mortality in seabream larvae at both ages, being higher for the youngest ones and with RGNNV/SJNNV. While 37 dph larvae survival greatly dropped from 1 dpi and remained low until 4 dpi the mock-infected group died from 2 to 6 dpi. Survival curves showed significance between mock-infected and RGNNV/SJNNV and SJNNV/RGNNV groups. For the group infected with the RGNNV/SJNNV genotype, this mortality is attributable to the virus at 1 dpi, due to the viral infection. Surprisingly, for SJNNV/RGNNV, this was not detected until 4 dpi, suggesting viral infection and replication is less effective or lower compared to RGNNV/SJNNV. On the other hand, in 86 dph larvae, mortality was lower than in 37 dph larvae, but significantly increased by both NNV reassortants compared with the mock-infected control. This manuscript represents the first description of the infective capabilities of SJNNV/RGNNV reassortants in seabream. Regarding previous studies with the RGNNV/SJNNV reassortant, clinical signs and/or mortalities higher than 80% occurred in 17–30 dph seabream larvae, but not in those of 35 or 75 dph [15], while mortality of 95% was achieved after 3–4 dpi in 20–25 dph larvae [14]. This fast mortality is in agreement with that observed in our study indicating the great susceptibility at these early ages, in which the management and production conditions seem to play a crucial role. In fact, restart of the disease and mortalities in survivors are associated with stress situations. In support of this mortality, we confirmed the NNV presence in the infected fish by the expression of the viral genes *rdrp* and *cp*, which always increased with the infection time; this had the highest result for the RGNNV/SJNNV genotype, and achieved higher expression levels in 37 dph larvae. The increase of the *rdrp* transcription was >4 logs during the infection with RGNNV/SJNNV in 37 dph larvae, similar to previous data [15], whereas it was around 1 log in 86 dph larvae, being related to the low mortality at this age. For SJNNV/RGNNV genotype, *rdrp* transcription increased >2 logs in 37 dph across the infection, while it was 1 log in 86 dph larvae. Thus, lower expression of viral genes in 86 dph larvae correlated with lower levels of mortalities for both reassortants. Interestingly, transcription of the RNA1 segment in both genotypes was higher than the transcription of the RNA2, as seen in previous investigations [15,16]. The increased pathogenicity of RGNNV/SJNNV and SJNNV/RGNNV genotypes compared with RGNNV may be due to several changes in RNA1 and RNA2 segments. Although RNA1-RGNNV has been shown to exhibit better replication capacity than RNA1-SJNNV, the presence of three transmembrane domains in the RNA1 of both reassortants was confirmed and this issue could improve the formation of the viral replication complex at 25 °C, the optimal temperature for seabream development [8,19,20], and support the replicative capacity of both reassortants in seabream larvae. Regarding the relation of the RNA2 segment with the viral pathogenicity, it has been found that two amino acids at positions 247 and 270 of the viral capsid of the RGNNV/SJNNV genotype modulate viral infectivity [6,21], causing a decrease in virulence in Senegalese sole and turbot (*Scophthalmus maximus*) [22,23]. These changes have been suggested to increase the affinity of the viral particle for the cellular receptor [13], though this characterization awaits confirmation for the SJNNV/RGNNV reassortant. In addition, isolation of infective particles was only achieved in 37 dph larvae infected with both reassortants, reaching higher titers in those infected with RGNNV/SJNNV, in agreement with previous data [14]. The fact of the impossibility to recover infective viral particles in the 86 dph larvae may be because of the low level of RNA obtained for the viral genes, which is indicative of the very low viral progeny formation. Therefore, our data about susceptibility, viral replication and titers are clearly in agreement with the literature and demonstrate that infectivity decreases with age, and that RGNNV/SJNNV is the most pathogenic for seabream larvae, though the impact of SJNNV/RGNNV is newly described. Further research linking genome/structural changes caused in the reassortants genotypes is needed to understand the increased virulence for seabream larvae.

In order to further investigate how RGNNV/SJNNV and SJNNV/RGNNV infection progressed in gilthead seabream larvae, some immune parameters were evaluated. Although our results failed to find great changes in the transcription upon NNV reassortant infection, as previously observed [16], we found important correlations in the gene profiles, and the major impact of larval age. First, our data show great correlation between the chaperone *hsp70*, antiviral (*mx* and *mda5*) and CMC (*nccrp1*, *prf*, *nkl*, *gzma* and *gzmb*) genes, which also show a major presence in the PCA. In addition, the PCA clearly identified a different transcription profile in the two ages, which is consistent with the viral replication, isolation and mortality observed in this study and others. Thus, gene expression was analysed separately for the two larval ages and hierarchical clustering analysis identified two different groups. Focusing on the different genes, our data showed slight and non-significant modification of the *hsp70* transcription, probably due to their high levels in mock-infected larvae as consequence of the handling stress. Hsp70 belongs to the heat-shock protein (HSP) family, whose members are largely up-regulated upon infection with RGNNV [24] and RGNNV/SJNNV [25]. These HSPs might serve as membrane receptors to internalize NNV particles, reduce the inflammation to favor viral replication and spread, and even interact with NNV proteins [26,27]. It is unclear whether this increase in the chaperones has a positive or negative role, since they have been implicated both as possible antivirals [28,29] and as factors favoring viral replication [30,31]. We then analysed the antiviral genes *mx* and *mda5*, *mx* being the canonical marker for the type I IFN response. RGNNV/SJNNV provoked an increase in expression of *mx* at 1 dpi in 37 dph larvae, which decreased later and was not maintained. However, against SJNNV/RGNNV, and in 86 dph larvae against both reassortants, expression of these two antiviral genes increases over infection and/or persists for days, suggesting that the IFN pathway is more effective in fighting infection in these cases. A transcriptomic study using RGNNV/SJNNV in 21 dph seabream larvae showed that the IFN pathway was deregulated, and this could favor viral evasion of the immune system, increasing its replication [16]. In contrast, the RGNNV genotype induced higher mRNA levels of *mx* resulting in much lower mortality [10], hence a correlation between *mx* levels and the pathogenic capacity of viruses is observed. Therefore, type I IFN antiviral response, mainly based on Mx production, might be orchestrating the susceptibility/resistance to NNV reassortants.

The importance of inflammation and NNV infection has also been studied [11,32,33]. In fact, production of the pro-inflammatory cytokine IL-1β has been pointed out as one of the key factors contributing to NNV disease since it directly drives neuronal and glia cell death, while its inhibition reduces NNV-associated death [34]. This is also suggested by our previous finding showing that RGNNV induces *il1b* transcription in the brain of European sea bass, which are very susceptible, and not in gilthead seabream, which are completely resistant [35]. Therefore, our data are in line with these observations since *il1b* increments were observed in infected 37 dph larvae, which showed great mortality, while *il1b* down-regulations occurred in 86 dph, in which very low mortalities were observed. We also focused on the CMC response, including the genes *nccrp1*, *prf*, *nkl*, *gzma* and *gzmb*, which have been previously identified in seabream against RGNNV infection and related to fish protection [10,24,36]. In 37 dph larvae, CMC genes cluster together and are slightly increased upon infection with RGNNV/SJNNV at 1 dpi and then drastically dropped, while increasing with the infection time for those infected with SJNNV/RGNNV. Reduction in the granzymes and perforin might lead to decreased elimination of RGNNV/SJNNV in 37 dph larvae as these molecules act against cells infected by intracellular pathogens, inducing their death by apoptosis [37,38,39]. Transcription of *gzma* was significantly up-regulated at 4 days after SJNNV/RGNNV infection but not *gzmb*. Similarly, GzmA seems to play a more important role than GzmB against RGNNV [24]. In addition, both *nkl* transcription and Nkl levels also increased upon SJNNV/RGNNV infection in young larvae but not upon RGNNV/SJNNV infection. This seems to indicate that RGNNV/SJNNV progression can inhibit the production of the CMC response to prevent the clearance of the host infected cells, thus continuing viral replication, as can be seen by the increase of viral genes up to 7 dpi. On the other hand, infection with SJNNV/RGNNV results in early depression of the CMC genes, but a later important increasing tendency, while in 86 dph larvae the impact of the CMC genes seems to be scarce. The increase of Nkl in 37 dph larvae infected with SJNNV/RGNNV reassortant also supports the idea of the involvement of the innate immune system in fighting viral infections of this genotype. In mammals, Nkl is produced by NK cells and T lymphocytes, so it is certain that in this case it is produced by NK-like cells, as the adaptative immune system is not yet fully developed at 37 dph in seabream but it is at 86 dph [40,41].

Finally, the decrease in *hamp* gene expression, a well-known AMP with anti-NNV function [42], also supports the idea that the RGNNV/SJNNV genotype decreases the activity of this particular arm of the innate immune system in order to survive and spread throughout the individual. Both proteins, Hamp and Nkl, were higher in 86 dph than 37 dph larvae, probably due to the presence of more cells producing them in the larger larvae.

## 4. Materials and Methods

### 4.1. Animals

Specimens of gilthead seabream (*Sparus aurata*) were bred at the *Centro Oceanográfico de Murcia, Instituto Español de Oceanografía* (IEO-CSIC) facilities from spawning culture broodstocks. For a month before the spawning period, the breeders were fed with commercial broodstock diets (Vitalis CAL diet from Skretting, Burgos, Spain). Fertilized eggs were harvested from superficial water, counted and placed in 0.5 m^3^ cylinder-conical tanks where they were incubated and hatching took place. The larval rearing was carried out under natural photoperiod and temperature using the “green water” technique in a 5000 L round tank with an initial density of about 60 eggs/L. Natural seawater (38‰ salinity) was heated to 17 ± 1 °C and filtered through mechanical and biological substrates. Water renewal was limited to 2% daily during the first 20 days of culture and was achieved by the addition of 70 mL/m^3^ of a microalgae concentrated solution (Phytobloom, Necton, Algarve, Portugal) containing 80% *Nannochloropsis oculata*. Subsequently, continuous water renewal (30%/h) and light aeration were provided in the tank. During the experiment, the light intensity was 1000 lux at the water surface, and the photoperiod was 16:8 (L:D). Larvae were successively fed with enriched rotifers (Selco, Inve Animal Health, Vigo, Spain) from 6 to 24 days post-hatching (dph), *Artemia nauplii* (Inve Animal Health) from 20 to 35 dph, enriched Instar II *Artemia* from 31 to 58 dph and a commercial dry pellet diet (Gemma Diamond diet from Skretting) from 54 dph onwards.

All specimens were handled in accordance with the Guidelines of the European Union Council (2010/63/EU) and the optimal zootechnical protocols of the *Centro Oceanográfico de Murcia* (IEO-CSIC, REGA ES300261040017), the Bioethical Committee of the IEO-CSIC and the approval of the Ministry of Water, Agriculture and Environment of the Autonomous Community Region of Murcia (Permit number A13200602).

### 4.2. Challenge with Nodavirus and Sampling

NNV reassortants (RGNNV/SJNNV, isolate 367.2.2005; and SJNNV/RGNNV, isolate 389/I96; kindly donated by Anna Toffan, IZSVe, Legnaro, Italy) were propagated in the E-11 cell line as elsewhere [43]. NNV stocks were titrated and the viral dilution infecting 50% of the cell cultures (TCID_50_/mL) calculated [44].

Seabream at two larval ages, 37 and 86 dph, were transferred to the infection room with tightly controlled input and outputs. Larvae were always randomly distributed in 9 tanks, forming 3 groups in triplicates, with independent recirculation water systems composed of a mechanical and biological filter, two aerators, a stabilization tank and a submersible recirculation pump. Nine-hundred seabream larvae of 37 dph were disposed into nine tanks at high density (3 larvae/mL) for 10 min to perform the challenge with 10^4^ TCID_50_/mL, of either RGNNV/SJNNV or SJNNV/RGNNV, and then returned to the conditions with a density of 25 larvae/L. Larvae were fed once a day with enriched Instar II Artemia. In a second trial, 270 seabream larvae of 86 dph were disposed into 9 tanks with a high density (60 larvae/L), infected as above and returned to a density of 0.5 larvae/L. In both trials, a mock-infected control group received the same amount of conditioned E-11 cell culture media at the same densities as the infected fish. All the experimental conditions of both experiments were performed in triplicate tanks.

Mortality was monitored daily and the cumulated percent of survival curves represented. Larvae were sampled at 1, 4 or 7 days post-infection (dpi), and also at 15 dpi in the case of the 86 dph larvae, snap frozen in liquid nitrogen and stored at −80 °C for later RNA isolation and gene expression, viral recovery or enzyme-linked immunosorbent assay (ELISA).

### 4.3. Gene Expression by Real-Time PCR (qPCR)

Whole larvae of 37 dph (*n* = 5), or the anterior third of 86 dph (*n* = 5) larvae, were individually placed in TRIzol Reagent (Thermo-Fisher Scientific, Waltham, MA, USA) and homogenized to isolate the total RNA as indicated by the manufacturer. One microgram of RNA was treated with DNase I to remove genomic DNA and the first strand of cDNA synthesized by the SuperScript IV Reverse Transcriptase (Thermo Fisher Scientific) with random hexamers.

The transcription levels of viral NNV RNA-dependent RNA polymerase (*rdrp*) and coat protein (*cp*), and gilthead seabream antiviral (*mda5* and *mx*), inflammatory (*il1b* and *il8*), stress (*hsp70*), cell-mediated cytotoxicity (CMC; *nccrp1*, *prf*, *nkl*, *gzma* and *gzmb*) and antimicrobial peptide (AMPs; *hamp* and *nkl*) genes were individually evaluated by real-time PCR with an ABI PRISM 7500 instrument (Applied Biosystems, MA, USA) using SYBR Green PCR Core Reagents (Applied Biosystems). Reaction mixtures were incubated for 10 min at 95 °C, followed by 40 cycles of 15 s at 95 °C, 1 min at 60 °C, and finally 15 s at 95 °C, 1 min 60 °C and 15 s at 95 °C. For each mRNA, gene expression was corrected by the *elongation factor 1-alpha* (*ef1a*) gene expression contents in each sample and expressed as 2^−∆Ct^, where ∆Ct is determined by subtracting the *ef1a* CT value from the target CT [45]. To ascertain that the NNV primers are able to identify the two reassortants, and allow comparisons, their efficiencies were determined using 10-fold dilution curves. The primers used were designed using the Oligo Perfect software tool (Thermo Fisher Scientific) and are shown in the Supplementary Table S2. Specificity of each primer pair was demonstrated using positive and negative samples and melting curves. Negative controls with no template were always included in the reactions.

### 4.4. Virus Isolation and Titration

Samples of pooled whole larvae of 37 dph (3 pools with 5 larvae each) at 1, 4 and 7 dpi, or the anterior third of 86 dph larvae (*n* = 2) at 15 dpi were used for viral isolation. Samples were homogenized in 10 volumes of phosphate buffer (PBS, pH 7.2) and the viral titer determined on E-11 cells after incubation of 10 days at 25°C [43,44]. Negative samples were titrated again.

### 4.5. Detection of Hamp and Nkl Proteins by ELISA

Specific detection of hepcidin antimicrobial (Hamp) and NK-lysin (Nkl) peptides were performed by an indirect ELISA [46]. To summarize, pooled whole 37 dph (5 pools with 5 larvae each) or anterior third (*n* = 2) 86 dph seabream larvae were homogenized in 10 volumes of 0.01 M PBS. The protein levels of the homogenates were analysed by the Bradford method [47]. Then, 40 μg of total proteins were diluted in coating buffer (50 mM Bicarbonate/Carbonate, pH 9.6) and incubated overnight at 4 °C in each well of flat bottom 96-well plates (Nunc, MA, USA). After incubation, the plate was washed with PBS containing 0.05% of Tween-20 (PBS-T) and subsequently blocked with 3% bovine serum albumin (BSA) in PBS for 1 h. Next, samples were incubated with the optimal dilution (1:2000 or 1:1000) of newly synthesized and validated rabbit immune sera against gilthead seabream Hamp or Nkl [46], respectively, for 1 h. Following that, samples were washed with PBS-T and incubated with anti-rabbit IgG-HRP (Thermo Fisher Scientific) at the optimal dilution of 1:1000 for 1 h. At the end, the reaction was revealed with 3,3′,5,5′-tetramethylbenzidine single solution (100 μL/well, Invitrogen) for 10 min and stopped with 50 μL of 2 M sulfuric acid. The absorbance was read at 450 nm with a plate reader (SPECTROstar, BMG). All assays were performed in duplicate. Hamp or Nkl synthetic peptides instead of samples were used as positive controls whilst samples without protein homogenates or primary antisera were used as negative controls.

### 4.6. Statistical Analysis

Data in figures are represented as mean ± standard error of the mean (SEM). Survival was presented by Kaplan–Meier curves and analysed by Log-rank (Mantel–Cox) test. Protein levels were analysed by a one-way analysis of variance (ANOVA) followed by Tukey’s *post-hoc* analysis. Non-parametric Pearson correlation test, ANOVA and a principal component analysis (PCA) were applied to test relations among gene expression, viral load, protein levels or survival. Statistical analysis and graphs were carried out using either SPSS or GraphPad software.

## 5. Conclusions

To summarize, the results obtained in this study corroborate the susceptibility of gilthead seabream larvae to infection by the RGNNV/SJNNV reassortant as well as its age-dependency. Very interestingly, this is the first report demonstrating that the SJNNV/RGNNV reassortant is also pathogenic for seabream larvae. High pathogenicity and viral replication of RGNNV/SJNNV in 37 dph larvae appears to be due to a general and time-decreasing tendency of the immune response. By contrast, the lower viral replication of SJNNV/RGNNV might be due to the opposite, the increasing immunity through time of infection, although not sufficiently to avoid mortalities. The differences in the survival between 37 and 86 dph larvae could be due to a more efficient antiviral response through Mx but also to a higher functional immunological maturity related to larval development and age. Further studies are needed to understand the vulnerability of gilthead seabream larvae to reassortant NNV genotypes and the involvement of the immune response.

## Figures and Tables

**Table 1:** Summary of the ANOVA results in 37 or 86 days post-hatching (dph) gilthead seabream larvae infected by immersion with RGNNV/SJNNV or SJNNV/RGNNV nodavirus (NNV) reassortants and sampled at 1, 4 or 7 days post-infection (dpi). Significant values (*p* < 0.05) are in bold. ANOVA, analysis of variance; *rdrp*, NNV RNA-dependent RNA polymerase; *cp*, NNV coat protein; *mx*, interferon-induced GTP-binding protein Mx; *mda5*, melanoma differentiation-associated protein 5; *hsp70*, heat-shock protein 70; *il1b*, interleukin 1 beta; *il8*, interleukin 8; *hamp*, hepcidin; *nccrp1*, non-specific cytotoxic cell receptor protein 1; *prf*, perforin; *gzma*, granzyme A; *gzmb*, granzyme B. ND, not determined due to insufficient groups.

Factor		dph	NNV	dpi	dph × NNV	dph × dpi	dpi × NNV	dph × dpi × NNV
Gene	*rdrp*	**0.028**	**0.008**	**0.009**	**0.011**	**0.011**	**0.001**	**0.002**
	*cp*	0.107	**0.003**	**0.003**	0.140	0.082	**0.000**	0.073
	*mx*	**0.000**	**0.036**	0.563	**0.036**	0.563	0.246	0.246
	*mda5*	**0.000**	0.805	0.278	0.791	0.297	**0.055**	**0.047**
	*hsp70*	**0.000**	0.533	0.875	0.516	0.872	0.547	0.545
	*il1b*	**0.000**	0.795	**0.000**	0.791	**0.000**	0.951	0.950
	*il8*	**0.001**	0.115	**0.007**	0.113	**0.007**	0.208	0.207
	*hamp*	0.817	0.122	0.266	0.607	0.656	0.441	0.556
	*nkl*	**0.000**	0.165	0.200	0.184	0.106	0.119	0.109
	*nccrp1*	**0.000**	0.168	**0.004**	0.165	**0.004**	0.081	0.082
	*prf*	**0.000**	0.112	0.923	0.323	0.314	**0.010**	**0.014**
	*gzma*	**0.000**	0.237	0.968	0.159	0.717	**0.005**	**0.012**
	*gzmb*	**0.000**	0.093	0.441	0.090	0.485	**0.044**	**0.047**
Protein	*Hamp*	ND	0.907	**0.007**	ND	ND	0.481	ND
	*Nkl*	ND	**0.004**	0.759	ND	ND	0.146	ND
Viral load		ND	**0.000**	**0.000**	ND	**0.000**	**0.000**	**0.000**
Survival		**0.000**	**0.000**	**0.000**	**0.000**	**0.000**	**0.001**	**0.000**

## Data Availability

Data are contained within the manuscript.

## Supplementary Materials

The following supporting information can be downloaded at: https://www.mdpi.com/article/10.3390/ijms23095092/s1.

## References

[1] Mori K.-I., Nakai T., Muroga K., Arimoto M., Mushiake K., Furusawa I. (1992). Properties of a new virus belonging to nodaviridae found in larval striped jack (*Pseudocaranx dentex*) with nervous necrosis. Virology.

[2] Iwamoto T., Mise K., Takeda A., Okinaka Y., Mori K.I., Arimoto M., Okuno T., Nakai T. (2005). Characterization of striped jack nervous necrosis virus subgenomic RNA3 and biological activities of its encoded protein B2. J. Gen. Virol..

[3] Nagai T., Nishizawa T. (1999). Sequence of the non-structural protein gene encoded by RNA1 of striped jack nervous necrosis virus. J. Gen. Virol..

[4] Bandín I., Souto S. (2020). Betanodavirus and VER disease: A 30-year research review. Pathogens.

[5] Toffolo V., Negrisolo E., Maltese C., Bovo G., Belvedere P., Colombo L., Valle L.D. (2007). Phylogeny of betanodaviruses and molecular evolution of their RNA polymerase and coat proteins. Mol. Phylogenet. Evol..

[6] Olveira J.G., Souto S., Dopazo C.P., Thiéry R., Barja J.L., Bandín I. (2009). Comparative analysis of both genomic segments of betanodaviruses isolated from epizootic outbreaks in farmed fish species provides evidence for genetic reassortment. J. Gen. Virol..

[7] Iwamoto T., Okinaka Y., Mise K., Mori K.-I., Arimoto M., Okuno T., Nakai T. (2004). Identification of host-specificity determinants in betanodaviruses by using reassortants between striped jack nervous necrosis virus and sevenband grouper nervous necrosis virus. J. Virol..

[8] Panzarin V., Cappellozza E., Mancin M., Milani A., Toffan A., Terregino C., Cattoli G. (2014). In vitro study of the replication capacity of the RGNNV and the SJNNV betanodavirus genotypes and their natural reassortants in response to temperature. Vet. Res..

[9] Castric J., Thiéry R., Jeffroy J., de Kinkelin P., Raymond J. (2001). Sea bream *Sparus aurata*, an asymptomatic contagious fish host for nodavirus. Dis. Aquat. Organ..

[10] Chaves-Pozo E., Guardiola F.A., Meseguer J., Esteban M.A., Cuesta A. (2012). Nodavirus infection induces a great innate cell-mediated cytotoxic activity in resistant, *Gilthead seabream*, and susceptible, European sea bass, teleost fish. Fish Shellfish Immunol..

[11] Moreno P., Lopez-Jimena B., Randelli E., Scapigliati G., Buonocore F., Garcia-Rosado E., Borrego J.J., Alonso M.C. (2018). Immuno-related gene transcription and antibody response in nodavirus (RGNNV and SJNNV)-infected European sea bass (*Dicentrarchus labrax* L.). Fish Shellfish Immunol..

[12] NaveenKumar S., Hassan M.A., Mahmoud M.A., Al-Ansari A., Al-Shwared W.K. (2017). Betanodavirus infection in reared marine fishes along the arabian gulf. Aquac. Int..

[13] Toffan A., Pascoli F., Pretto T., Panzarin V., Abbadi M., Buratin A., Quartesan R., Gijón D., Padrós F. (2017). Viral nervous necrosis in Gilthead sea bream (*Sparus aurata*) caused by reassortant betanodavirus RGNNV/SJNNV: An emerging threat for Mediterranean aquaculture. Sci. Rep..

[14] Volpe E., Gustinelli A., Caffara M., Errani F., Quaglio F., Fioravanti M.L., Ciulli S. (2020). Viral nervous necrosis outbreaks caused by the RGNNV/SJNNV reassortant betanodavirus in Gilthead sea bream (*Sparus aurata*) and European sea bass (*Dicentrarchus labrax*). Aquaculture.

[15] Toffan A., Biasini L., Pretto T., Abbadi M., Buratin A., Franch R., Dalla Rovere G., Panzarin V.M., Marsella A., Bargelloni L. (2021). Age dependency of RGNNV/SJNNV viral encephalo-retinopathy in Gilthead sea bream (*Sparus aurata*). Aquaculture.

[16] Peruzza L., Pascoli F., Dalla Rovere G., Franch R., Ferraresso S., Babbucci M., Biasini L., Abbadi M., Panzarin V., Toffan A. (2021). Transcriptome analysis reveals a complex response to the RGNNV/SJNNV reassortant nervous necrosis virus strain in sea bream larvae. Fish Shellfish Immunol..

[17] Chérif N., Thiéry R., Castric J., Biacchesi S., Brémont M., Thabti F., Limem L., Hammami S. (2009). Viral encephalopathy and retinopathy of *Dicentrarchus labrax* and *Sparus aurata* farmed in tunisia. Vet. Res. Commun..

[18] Moretti A., Pedini Fernández-Criado M., Cittolin G., Guidastri R. (1999). Manual on Hatchery Production of Seabass and Gilthead Seabream: Volume 1.

[19] Guo Y.X., Chan S.-W., Kwang J. (2004). Membrane association of greasy grouper nervous necrosis virus protein A and characterization of its mitochondrial localization targeting signal. J. Virol..

[20] Mézeth K.B., Nylund S., Henriksen H., Patel S., Nerland A.H., Szilvay A.M. (2007). RNA-dependent RNA polymerase from Atlantic halibut nodavirus contains two signals for localization to the mitochondria. Virus Res..

[21] Souto S., Mérour E., Biacchesi S., Brémont M., Olveira J.G., Bandín I. (2015). In vitro and in vivo characterization of molecular determinants of virulence in reassortant betanodavirus. J. Gen. Virol..

[22] Souto S., Olveira J.G., Dopazo C.P., Bandín I. (2016). Reassortant betanodavirus infection in turbot (*Scophthalmus maximus*). J. Fish Dis..

[23] Souto S., Olveira J.G., Alonso M.C., Dopazo C.P., Bandín I. (2018). Betanodavirus infection in bath-challenged *Solea senegalensis* juveniles: A comparative analysis of RGNNV, SJNNV and reassortant strains. J. Fish Dis..

[24] Chaves-Pozo E., Valero Y., Lozano M.T., Rodríguez-Cerezo P., Miao L., Campo V., Esteban M.A., Cuesta A. (2019). Fish granzyme A shows a greater role than granzyme B in fish innate cell-mediated cytotoxicity. Front. Immunol..

[25] Vázquez-Salgado L., Olveira J.G., Dopazo C.P., Bandín I. (2021). Effect of rearing density on nervous necrosis virus infection in senegalese sole (*Solea senegalensis*). J. Fish Dis..

[26] Chang J.-S., Chi S.-C. (2015). GHSC70 is involved in the cellular entry of nervous necrosis virus. J. Virol..

[27] Lu M.W., Ngou F.H., Chao Y.M., Lai Y.S., Chen N.Y., Lee F.Y., Chiou P.P. (2012). Transcriptome characterization and gene expression of *Epinephelus* spp. in endoplasmic reticulum stress-related pathway during betanodavirus infection in vitro. BMC Genomics.

[28] De La Vega E., Hall M.R., Degnan B.M., Wilson K.J. (2006). Short-term hyperthermic treatment of *Penaeus monodon* increases expression of heat shock protein 70 (HSP70) and reduces replication of gill associated virus (GAV). Aquaculture.

[29] Lin Y.-R., Hung H.-C., Leu J.-H., Wang H.-C., Kou G.-H., Lo C.-F. (2011). The role of aldehyde dehydrogenase and Hsp70 in suppression of white spot syndrome virus replication at high temperature. J. Virol..

[30] Pham P.H., Sokeechand B.S.H., Hamilton M.E., Misk E., Jones G., Lee L.E.J., Lumsden J.S., Bols N.C. (2019). VER-155008 induced Hsp70 proteins expression in fish cell cultures while impeding replication of two RNA viruses. Antiviral Res..

[31] Shan L.P., Chen X.H., Ling F., Zhu B., Wang G.X. (2018). Targeting Heat Shock Protein 70 as an antiviral strategy against grass carp reovirus infection. Virus Res..

[32] Poisa-Beiro L., Dios S., Ahmed H., Vasta G.R., Martínez-López A., Estepa A., Alonso-Gutiérrez J., Figueras A., Novoa B. (2009). Nodavirus infection of sea bass (*Dicentrarchus labrax*) induces up-regulation of galectin-1 expression with potential anti-inflammatory activity. J. Immunol..

[33] Poisa-Beiro L., Dios S., Montes A., Aranguren R., Figueras A., Novoa B. (2008). Nodavirus increases the expression of Mx and inflammatory cytokines in fish brain. Mol. Immunol..

[34] Chiang Y.H., Wu Y.C., Chi S.C. (2017). Interleukin-1β secreted from betanodavirus-infected microglia caused the death of neurons in giant grouper brains. Dev. Comp. Immunol..

[35] Valero Y., Arizcun M., Esteban M.Á., Bandín I., Olveira J.G., Patel S., Cuesta A., Chaves-Pozo E. (2015). Nodavirus colonizes and replicates in the testis of *Gilthead seabream* and European sea bass modulating its immune and reproductive functions. PLoS ONE.

[36] Valero Y., Chaves-Pozo E., Cuesta A. (2020). NK-Lysin is highly conserved in European sea bass and *Gilthead seabream* but differentially modulated during the immune response. Fish Shellfish Immunol..

[37] Ewen C.L., Kane K.P., Bleackley R.C. (2012). A quarter century of granzymes. Cell Death Differ..

[38] Voskoboinik I., Whisstock J.C., Trapani J.A. (2015). Perforin and granzymes: Function, dysfunction and human pathology. Nat. Rev. Immunol..

[39] Praveen K., Leary J.H., Evans D.L., Jaso-Friedmann L. (2006). Nonspecific cytotoxic cells of teleosts are armed with multiple granzymes and other components of the granule exocytosis pathway. Mol. Immunol..

[40] Cordero H., Guzmán-Villanueva L.T., Chaves-Pozo E., Arizcun M., Ascencio-Valle F., Cuesta A., Esteban M.A. (2016). Comparative ontogenetic development of two marine teleosts, *Gilthead seabream* and European sea bass: New insights into nutrition and immunity. Dev. Comp. Immunol..

[41] Mulero I., Sepulcre M.P., Fuentes I., García-Alcázar A., Meseguer J., García-Ayala A., Mulero V. (2008). Vaccination of larvae of the bony fish *Gilthead seabream* reveals a lack of correlation between lymphocyte development and adaptive immunocompetence. Mol. Immunol..

[42] Chia T.J., Wu Y.C., Chen J.Y., Chi S.C. (2010). Antimicrobial peptides (AMP) with antiviral activity against fish nodavirus. Fish Shellfish Immunol..

[43] Iwamoto T., Mise K., Mori K.I., Arimoto M., Nakai T., Okuno T. (2001). Establishment of an infectious RNA transcription system for striped jack nervous necrosis virus, the type species of the betanodaviruses. J. Gen. Virol..

[44] Reed L.J., Müench H. (1938). A simple method of estimating fifty per cent endpoints. Am. J. Epidemiol..

[45] Pfaffl M.W., Dorak T. (2007). Relative Quantification. Real-Time PCR.

[46] Cervera L., González-Fernández C., Arizcun M., Cuesta A., Chaves-Pozo E. (2022). Severe natural outbreak of *Cryptocaryon irritans* in *Gilthead seabream* produces leukocyte mobilization and innate immunity at the gill tissue. Int. J. Mol. Sci..

[47] Bradford M. (1976). A rapid and sensitive method for the quantitation of microgram quantities of protein utilizing the principle of protein-dye binding. Anal. Biochem..

