# Supplementary material for: Profile of Innate Immunity in Gilthead Seabream Larvae Reflects Mortality upon Betanodavirus Reassortant Infection and Replication
Source: Int J Mol Sci. 2022 May 3;23(9):5092. doi: 10.3390/ijms23095092 (PMC9105140; doi:10.3390/ijms23095092)
Supplement: Supplementary file 1 [file ijms-23-05092-s001.zip › ijms-1695428-supplementary.pdf]

Table S1: Data of the principal component analysis with the Varimax rotation method. Correlations higher than 0.5 are in bold.

		Component	
		1	2
Gene	*rdrp*	-0.028	**0.687**
	cp	-0.053	**0.659**
	mx	**0.946**	-0.068
	mda5	**0.924**	0.199
	hsp70	**0.933**	0.106
	il1b	0.414	**0.511**
	il8	0.261	**0.58**
	nccrp1	**0.750**	0.405
	hamp	0.289	**-0.67**
	nkl	**0.874**	-0.253
	prf	**0.901**	-0.254
	gzma	**0.901**	0.194
	gzmb	**0.964**	-0.055
Survival		**-0.746**	**-0.535**

Table S2: Primers used for real-time PCR analysis.

Gene name	Gene abbreviation	Accession number	Primer sequence (5′ → 3′)
Nodavirus RNA-dependent RNA polymerase	*rdrp*	AF319555	GTGTCCGGAGAGGTTAAGGATG<br>CTTGAATTGATCAACGGTGAACA
Nodavirus coat protein	*cp*	D38636	CAACTGACAACGATCACACCTTC<br>CAATCGAACACTCCAGCGACA
Elongation factor 1 alpha	*ef1a*	AF184170	CTTCAACGCTCAGGTCATCAT<br>GCACAGCGAAACGACCAAGGGGA
Interferon-induced GTP-binding protein Mx	*mx*	FJ490556<br>FJ490555<br>FJ652200	AAGAGGAGGACGAGGAGGAG<br>CATCCCAGATCCTGGTCAGT
Melanoma differentiation-associated protein 5	*mda5*	HS988207	CATCGAGATCATCGAGGACA<br>CCAGATGTCGCTCTTGAAGG
Interleukin 1 beta	*il1b*	AJ277166	GGGCTGAACAACAGCACTCTC<br>TTAACACTCTCCACCCTCCA
Interleukin 8	*il8*	AM765841	GCCACTCTGAAGAGGACAGG<br>TTTGGTTGTCTTTGGTCGAA
Heat-shock protein 70	*hsp70*	EU805481	AATGTTCTGCGCATCATCAA<br>GCCTCCACCAAGATCAAAGA
Non-specific cytotoxic cell receptor protein 1	*nccrp1*	AY651258	ACTTCCTGCACCGACTCAAG<br>TAGGAGCTGGTTTTGGTTGG
Granzyme A	*gzma*	MK568066	GCTGCTCGGAGTCACTTCTT<br>GGATCCAGGTGAGCTGCTTT
Granzyme B	*gzmb*	AM957224	GAAACAAAGGAACGGTCAA<br>GAGCTGTCCATCTTTTGCTTG
Perforin	*prf*	AM950868	CAGACCATGAGGGGAACACT<br>TGGAACCATGCAACACCTTA
NK-lysin	*nkl*	MN240490	CGCACCTCGGAGAACTGATT<br>TCCACGTCGCTTCGGTAAAA
Hepcidin	*hamp*	CB184616	GCCATCGTGCTCACCTTTAT<br>CTGTTGCCATACCCCATCTT
